# Assessment of Acoustic Voice Parameters After Anterior Cervical Discectomy and Fusion

**DOI:** 10.7759/cureus.20611

**Published:** 2021-12-22

**Authors:** Abdurrahman B Cengiz, Ebru Doruk

**Affiliations:** 1 Otorhinolaryngology, Bagcilar Training and Research Hospital, Istanbul, TUR; 2 Neurosurgery, Bagcilar Training and Research Hospital, Istanbul, TUR

**Keywords:** acoustic analysis, otolaryngologist, spine surgery, vocal cord paralysis, acdf

## Abstract

Background

Anterior cervical discectomy and fusion (ACDF) is a surgical treatment approach for cervical spine diseases. Alteration in voice quality is a commonly encountered concern after perilaryngeal neck surgeries. Vocal cord paralysis is a known complication of ACDF. In this study, we aimed to investigate the effect of ACDF on acoustic voice parameters and to compare ACDF with posterior cervical discectomy and fusion (PCDF).

Methodology

In this study, we investigated 52 patients admitted to the hospital with symptoms related to cervical spinal cord compression and underwent spine surgery in the Neurosurgery Clinic (26 underwent ACDF and 26 underwent PCDF). For standardization, 25 healthy age and gender-matched volunteers were evaluated as the control group. The voices of the patients were analyzed digitally preoperatively and at first and third months postoperatively. As acoustic parameters, jitter, shimmer, basal frequency, and normalized noise energy were recorded. All patients were examined preoperatively and postoperatively for laryngeal pathology and were asked to fill the Voice Handicap Index-10 (VHI-10).

Results

The changes in four of the five acoustic parameters from baseline to postoperative first-month assessment in the ACDF group were significant (p < 0.05). These parameters almost approached normal values in the analysis performed at three months. In the PCDF group, no significant differences were seen in the acoustic analysis of the patients in comparison to the preoperative and the first and third-month assessments. The VHI-10 values were not significantly different among the patients who underwent ACDF or PCDF or control patients at any postoperative time point.

Conclusions

Our study demonstrated that voice parameters in patients who underwent ACDF worsened significantly after the surgery compared with patients who underwent PCDF; however, these changes recovered within three months postoperatively. The possible causes for these findings include the retraction of the vagus and the recurrent laryngeal nerve, postoperative edema of strap muscles, intubation trauma to the vocal folds, and other laryngeal structures.

## Introduction

Anterior cervical discectomy and fusion (ACDF) is the most widely used vertebral instrumentation procedure for the treatment of symptomatic cervical degenerative disc diseases [1]. This procedure provides near-normal correction of the biomechanical structure and allows direct decompression of the spinal cord and foramen [2]. An alternative posterior approach (through the back of the neck) that involves removing all or part of a damaged disc to relieve spinal cord or root pressure is called posterior cervical discectomy and fusion (PCDF) [3]. Although ACDF is successful and safe, complications may occur during or after the procedure due to the adjacent anatomic structures in the anterior neck [4]. Complications include tracheal or esophageal perforation, internal jugular vein injury, carotid or vertebral artery injury, and neural damage to the sympathetic or cervical nerve roots or the vagal nerve and its branch the recurrent laryngeal nerve (RLN) [5]. The RLN on the right side that originates from the right vagus nerve loops around the brachiocephalic artery and ascends in the right tracheoesophageal groove, entering the larynx from behind the cricothyroid joint. Vocal fold paralysis (VFP) secondary to RLN injury is the most complication associated with this procedure [6]. However, hoarseness can occur due to subclinical paralysis which is called paresis. RLN injury is not the only cause of voice alteration because many patients notice minimal changes immediately after operation without any evidence of RLN damage. The relationship between the surgical procedure and voice changes has not been fully elucidated. There may be other factors leading to voice changes, such as injury of the superior laryngeal nerve (SLN) and pre-thyroid strap muscles. This prospective study aimed to evaluate the effect of ACDF and compare it with PCDF along with voice quality and correlate any change to the extent of surgical dissection.

## Materials and methods

In this prospective study, we investigated 52 patients who were admitted to the hospital with symptoms related to cervical spinal cord compression and underwent spine surgery in the Neurosurgery Clinic at the University of Medical Sciences, Bagcilar Education and Research Hospital between December 2019 and December 2020. Overall, 26 patients underwent ACDF and the remaining 26 underwent PCDF. In this study, 25 healthy age and gender-matched volunteers were examined as the control group. The inclusion criteria included healthy phonation and vocal functions. Exclusion criteria included a history of a voice disorder or recent subjective voice problems, a history of voice therapy, any other disease that might affect normal voice function, a history of any otolaryngologic operation or recent abnormality, and a history of respiratory tract infection in the last three weeks before evaluation. Patients with a history of cranial, spinal cord, laryngeal, or neck surgery were also excluded from the study. Further, patients with vocal cord paralysis after the surgery were excluded. Study approval was obtained from the ethics committee and patients were informed about the study goals.

Surgical protocols

Information outlining the nature of the surgery, including the side of the approach and levels of the cervical spine, duration of the surgery, intraoperative complications, and patient demographics were collected. A single experienced consultant neurosurgeon performed all the operations under general anesthesia through endotracheal intubation with an appropriate size and low cuff pressure. The surgeon employed the same surgical technique based on the dissection of the tissues to prevent any injury to the vagus nerve and RLN in the operating field. In the anterior procedure, the Smith-Robinson [7] approach was employed which involved exposing the front of the spine, along with retracting crucial organs such as the carotid artery, internal jugular vein, esophagus, trachea, superior thyroid artery, vagal and recurrent nerve, and sympathetic trunk.

Voice analysis

The routine laryngeal examination was performed on all patients before the surgery and four and twelve weeks after the surgery by an otolaryngologist. The evaluation included a video laryngoscopy and laryngostroboscopy evaluation. The movement ability and the shape of the vocal cords were recorded. Voice recordings of the patients were performed before the operation and four and twelve weeks after the spine surgery. The patients were examined in a sound-treated room for healthy recording. They were asked to sit in front of a table with a microphone (C01U Pro Microphone, Samson, Frankfort, Germany) at a distance of 20 cm from the mouth, and they were asked to phonate a vowel /i/ and /a/ in habitual pitch and loudly for at least five seconds and read a standard text named “Diet” in a silent room (less than 50 dB noise). Voice samples were recorded directly in Praat® software. At least three voice samples were digitally recorded on a personal computer in a phoniatry laboratory. All study participants were prepared to voice a vocal sample at a conversational voice intensity between 50 and 60 dB. The perturbation of frequency was measured as jitter, and the perturbation of amplitude was measured as shimmer. The vocal parameters analyzed with Praat® included jitter (ddp, ppq5, rap, local) shimmer (local), pitch (median, mean, minimum, and maximum), number of pulses and periods, shimmer (apq3, apq5, apq11, dda), and mean harmonics-to-noise ratio (HNR).

In addition, the Turkish version of the Voice Handicap Index-10 (VHI-10) was used to quantify patients’ perception of their voice handicap, as validated by Kiliç et al. [8]. VHI-10 includes 10 questions, with each item scored from 0 to 4 (0 = none and 4 = always). The results of the questionnaire ranged between 0 and 40 points.

Statistical analysis

The Statistical Package for the Social Sciences (SPSS) version 20.0 (IBM Corp., Armonk, NY, USA) was used in the analysis. The paired-sample t-test was used to correlate normally distributed preoperative and postoperative data. The Student’s t-test and Mann-Whitney U test were used for normally and non-normally distributed data, respectively. The Wilcoxon test was used to compare non-normally distributed parameters. P-values of <0.05 were considered statistically significant.

## Results

A total of 63 patients were enrolled in the study, but 11 were excluded due to abnormal findings in the preoperative laryngologic examination. Four patients had vocal cord pathology, six had a history of otolaryngologic operations (three underwent a septoplasty, two underwent a tonsillectomy, and one underwent a rhinoplasty), and one patient had RLN paralysis on the right side. The average age of patients was 52 (30-72) years, and the male-to-female ratio was similar (males: 48%, females: 52%). The characteristics of the patients included in the study are presented in Table 1. The average duration of ACDF was 135 (90-215) minutes.

**Table 1:** Characteristics of the study population. ACDF: anterior cervical discectomy and fusion; PCDF: posterior cervical discectomy and fusion; NS: non-calculated; C: cervical vertebra

		Number (%)	P-value
Gender	Male	25 (48%)	0.786
	Female	27 (52%)	
Operation type	ACDF	26 (50%)	NS
	PCDF	26 (50%)	
Operated cervical spine segments	C2-C3, C3-C4	18 (34.6%)	>0.05
	C4-C5, C5-C6	20 (38.4%)	
	C6-C7, C7-T1	14 (26.9%)	

The mean length of the incision was 4 (3.2-6.7) cm. Overall, 18 (34.6%) patients were operated from high spinal segments, 20 (38.4%) patients were operated from middle segments, and 14 (26.9%) patients underwent low approach spinal surgery. Characteristics of the healthy individuals are presented in Table 2. There was no statistical difference regarding age and gender between the study and control groups.

**Table 2:** Features and acoustical parameters of 25 healthy participants. SDF0: standard deviation of the fundamental frequency; NNE: normalized noise energy; VHI-10: Voice Handicap Index-10

Parameters	Mean values	Range
Age (years)	52.2	30–72
Jitter (%)	0.31	0.23–0.36
Shimmer (%)	2.11	1.25–5.18
SDF0 (Hz)	1.29	1.11–2.13
NNE (dB)	-12.21	-13.13–-7.21
VHI-10 value	2.1	1–8

The recorded acoustic parameters, namely, the standard deviation of the fundamental frequency (SDF0), jitter, shimmer, and normalized noise energy (NNE), of the two groups preoperatively and at one and three months postoperatively are presented in Table 3. Alteration of voice quality one month following the operation was only noted in the ACDF group. In the PCDF group, no statistically significant difference was noted in any of the recorded acoustic parameters at one month postoperatively. In the ACDF group, amplitude perturbation (shimmer) significantly increased from 2.15% to 3.88% (p = 0.012). Similarly, frequency perturbation (jitter) significantly increased from 0.32% recorded before the operation to 1.21% one month after the surgery (p = 0.009). Differences in the NNE ratio in the ACDF group were statistically significant according to the first-month analysis.

**Table 3:** Comparison of acoustic voice parameters in the ACDF and PCDF groups. Statistical analysis (Wilcoxon signed-rank test, two-sided). P-value of <0.05 is statistically significant. *Comparison between preoperative and postoperative first-month values; **comparison between preoperative and postoperative third-month values. ACDF: anterior cervical discectomy and fusion; PCDF: posterior cervical discectomy and fusion; SD: standard deviation; SDF0: standard deviation of the fundamental frequency; NNE: normalized noise energy; VHI-10: Voice Handicap Index-10

	Acoustic parameter	Preoperative	Postoperative (1 month)	P-value*	Postoperative (3 months)	P-value**
		Mean ± SD	Mean ± SD		Mean ± SD	
ACDF group	Jitter (%)	0.32 ± 0.13	1.21 ± 1.11	0.009	0.62 ± 0.36	0.677
	Shimmer (%)	2.15 ± 1.65	3.88 ± 1.05	0.012	2.72 ± 1.12	0.333
	SDF0 (Hz)	1.59 ± 1.32	1.84 ± 1.48	0.008	1.93 ± 1.43	0.567
	NNE (dB)	-12.21 ± -7.21)	-9.64 ± -3.79	0,008	-12.43 ± -6.67	0.09
	VHI-10 value	2.4 ± 2.0	2.5 ± 1.8	0.789	2.5 ± 2.0	0.10
PCDF group	Jitter (%)	0.12 ± 0.10	0.19 ± 0.16	0.095	0.18 ± 0.17	0.098
	Shimmer (%)	2.19 ± 0.72	2.64 ± 1.62	0.088	2.64 ± 1.25	0.095
	SDF0 (Hz)	1.19 ± 0.61	1.29 ± 1.24	0.123	1.28 ± 0.94	0.177
	NNE (dB)	-12.25 ± -6.76	-12.75 ± -9.41	0.235	-12.16 ± -7.45	0.856
	VHI-10 value	2.5 ± 2.2	2.6 ± 2.0	0.722	2.4 ± 1.6	0.944

As shown in Table 3, the acoustic analysis showed that there were no significant differences in NNE values between the preoperative and postoperative third-month recordings in the ACDF group (p = 0.09). Similarly, significant differences were not noted in jitter or shimmer values (p = 0.677 and 0.333, respectively). Finally, the changes in acoustic parameters from baseline to the postoperative first-month assessment in the ACDF group were significant. However, these findings almost approached normal values in the analysis performed at three months. In the PCDF group, there was no statistically significant difference between the preoperative and the first and third-month postoperative acoustic parameters. In conclusion, all acoustic parameters significantly worsened in the early period after ACDF but not after PCDF. In contrast, the VHI-10 scores were not significantly different among patients who underwent ACDF or PCDF or controls at any postoperative time point.

## Discussion

Noticeable hoarseness as a complication of anterior cervical spine surgery has been well documented in the literature [9]. The etiology of vocal cord paralysis following ACDF relates to the sectioning or retraction injury of the RLN. SLN (particularly in high approaches) may also be impacted due to injury, even though it is not well documented in the literature. Unilateral VFP usually presents as hoarseness, breathy voice, aspiration, and dysphagia. On the other hand, bilateral VFP usually presents as respiratory distress and stridor which requires emergent treatment [6,10].

Frequently, RLN exposure is not necessary during anterior cervical spine surgery. Because the course of this thin nerve (1-3 mm) varies, the surgeon may experience difficulty in identifying and exposing the nerve. In thyroid surgery, surgeons usually expose the RLN to avoid injury in contrast to ACDF. According to a previous study, the incidence of permanent and temporary RLN palsy varies between 7% and 11% [11]. In our study, right RLN palsy occurred in one of the 26 patients (3.8%), which is consistent with the literature. Recently, it has been shown that multidisciplinary ACDF procedures, with the help of head and neck surgeons employing the preservative approach to protect RLN, potentially reduce the rates of dysphagia [12]. Despite otolaryngologists who may have never performed ACDF, training in neck dissection, laryngectomy, and thyroid surgeries prepare surgeons for challenging dissections, including revisions, patients with neck ankylosis, and post-radiation. This may explain why the multidisciplinary approach in ACDF reduces the incidence of RLN palsy [13].

In this study, we investigated that, in the absence of any apparent nerve injury, there may be complaints of voice alteration or hoarseness in patients following ACDF surgery. Hence, we performed the acoustic analysis to evaluate voice-related disorders and post-surgery voice outcome assessments. The possible causes of hoarseness in patients who receive general anesthesia include vocal cord and arytenoid cartilage injury resulting from endotracheal intubation. In one study, endotracheal tube cuff pressure and intraoperative laryngeal electromyographic recordings were compared. The study found that patients with postoperative hoarseness were more likely to have increased intraoperative cuff pressure and decreased electromyographic activity [14]. Hamdan et al. suggested that all vocal symptoms subsided with no significant difference to baseline values at 24 hours postoperatively and that the acoustic parameters did not change significantly at one month [15]. Although several studies have reported RLN injury after ACDF, this is the first study that evaluated specific acoustic parameters of patients after both anterior and posterior spine surgeries. This study has shown that the patients’ voice functions may be affected after ACDF compared with PCDF Acoustic analysis demonstrates that voice problems are significant and very common in the first month after ACDF. However, in the PCDF group, voice quality is not affected in the first and third months postoperatively.

Computerized acoustic analysis is a very useful technique for detecting voice disorders objectively. The shimmer test assesses the variations of intensity. The jitter test indicates pitch variability and expresses changes in the periodicity of the fundamental frequency (F0). For the assessment of the voice quality, jitter and shimmer are frequently used for detecting irregularity of basic pitch and amplitude in the acoustic signal, as well as changes in the shape, length, or stiffness of the vocal cords. According to our study, the PCDF group showed no significant changes regarding the acoustic values at the first and third months postoperatively compared with the preoperative values. However, in the ACDF group, shimmer, jitter, NNE, and mean Fo values significantly worsened at the first month postoperatively compared with the preoperative values; however, the values improved and approached preoperative values within three months. These results showed that the effect on laryngeal functions of manipulations made during the anterior surgery lasted for approximately one month but disappeared within three months. Voice alteration is a frequent complication of ACDF that usually recovers over time. Erwood et al. evaluated 67 patients who had ACDF and found vocal cord paralysis in 3% of the patients. The swallowing disorder was reported in 13% of the patients. A meta-analysis reported an incidence of 14.1% of permanent vocal cord paralysis. Winslow et al. reported high rates of postoperative dysphagia and dysphonia of 60% and 51%, respectively, in a large study including mostly revision operations [16]. Several studies have suggested that dysphagia and dysphonia symptoms resolve within two to three months of surgery and rarely persist longer than a year [17,18]. Yue et al. reported that singing disorder was seen in 21.6% of 176 patients postoperatively, occurring more frequently if the C3/4 disc surgery was performed and in patients who underwent a greater total number of anterior cervical surgeries. The presence of dysphonia was not related to smoking status, age, duration of the procedure, and the number of levels operated [19]. Values of the VHI-10 were not significantly different in patients who underwent ACDF or PCDF. These results showed that subjective changes in voice after spine surgery were minimal and unrecognizable by the patients. Furthermore, Jung et al. suggested that the left-sided approach in anterior cervical spine surgery and endotracheal cuff pressure reduction reduce the incidence of postoperative and irreversible vocal cord paralysis [20]. In this study, we measured vocal alterations in the early period after the ACDF operation. Surgical dissection of the SLN area and the superior thyroid artery, retraction of the vagal nerve and its branch RLN, and postoperative pain of strap muscles and perilaryngeal tissue were probable causes of the voice changes, although vocal cord movement examinations were normal.

## Conclusions

This study demonstrated that voice parameters in patients who underwent ACDF significantly changed following surgery compared with patients who underwent PCDF; however, these changes recovered within three months. The possible causes for these findings may be the retraction of the vagus and the RLN, postoperative edema of strap muscles, intubation trauma to the vocal folds, and other laryngeal structures.
